# Adapting to innovation: Reflections on electrophysiology training amid a transformative shift

**DOI:** 10.1016/j.hrcr.2024.11.001

**Published:** 2024-12-16

**Authors:** Chengyue Jin, Maryam Saleem, Jacob Koruth

**Affiliations:** Helmsley Electrophysiology Center, Department of Cardiology, Icahn School of Medicine at Mount Sinai, New York, NY

Pulsed-field ablation (PFA) is an innovative, non–thermal energy source that uses rapid, high-voltage electrical pulses to irreversibly electroporate as well as denature cell membranes, resulting in cell death. This energy source has been recognized as a significant advancement, offering a safer, more efficient, and effective method for performing atrial fibrillation (AF) ablation. Currently, 2 PFA catheters are approved for use in the United States; both of these are multi-electrode in design and primarily intended for pulmonary vein (PV) isolation rather than the focal, point-by-point approach used by traditional radiofrequency (RF) catheters.

Current PFA catheters are somewhat comparable to cryoablation catheters by virtue of an over-the-wire, PV-focused design and one can reasonably assume that the replacement of cryoballoon use by PFA is unlikely to change the skillset needed to operate these catheters. However, the impact of changes in the skillset required between RF catheters and PFA catheters is substantial. Several electrophysiology (EP) fellowship programs have already incorporated, or are in the process of acquiring, PFA technologies. This shift represents a pivotal change in fellowship training, especially from the perspective of the use of focal catheters—a skillset that requires time to develop and perfect. This is likely to have a significant impact on the training experience for current EP fellows, especially those who enter programs that are already using PFA as the primary strategy for AF procedures. This new trend in exposure to different catheters will only be amplified, as the number of PFA procedures is expected to rise and RF and cryoablations for PV isolation will likely decrease.

Considering these changes, concerns have emerged among current and prospective EP fellows that, with the shift to PFA, the ability to develop adequate skill levels for point-by-point catheter ablation will be adversely impacted. To assess the impact of this transition, we conducted a pilot online survey targeting current EP fellows and first-year attendings in the United States who experienced the shift to PFA during their training. The survey revealed that more than one-half of the respondents’ programs now perform more PFA procedures for AF than RF ablations, with this trend expected to continue to grow. Most fellows reported improvements in procedure time, workflow efficiency, and increases in case volume with PFA compared with when only RF or cryoablation was available. However, more than 60% expressed concerns that their training in point-by-point catheter ablation had been negatively impacted, highlighting the need for additional focused training for this technique. Although most fellows believe that PFA will become the dominant technology in AF ablation, they also foresee point-by-point RF ablation retaining importance, particularly for linear and focal ablations for either atrial flutters or supraventricular tachycardia.

Such transitions naturally introduce a degree of uncertainty with respect to fellows feeling adequately trained and can potentially affect fellowship training in both positive and negative ways. Concerns about losing exposure to certain traditional procedural skills as technological advances occur are not new. A similar situation occurred when RF-assisted transseptal tools were introduced, leading to a decline in the use of the Brockenbrough needle, which is now used only rarely in some fellowship programs. Although newer point-by-point PFA catheters with integrated mapping capabilities are under development, it is uncertain how successful these will be or whether there will be sufficient widespread use to ensure adequate exposure and training for all EP fellows.

Because PV isolation for AF ablation constitutes a large portion of fellowship procedural volume, a strategic approach is necessary to ensure fellows receive sufficient exposure to point-by-point catheter skills. RF will likely continue to have a role in certain cases, such as atrial flutter and redo AF ablations, and will provide valuable learning opportunities for fellows. The recognition of how important these cases may be for current fellows needs to be pointed out and potential discussion within programs may be needed so that these opportunities are capitalized on. In addition, simulators and animal laboratory training can serve as effective supplements. Finally, as discussed, should focal PFA catheters with point-by-point capabilities gain widespread use, we may eventually strike a balance between competing catheter designs, thus ensuring a comprehensive training experience for future electrophysiologists.

## Disclosures

The authors have no conflicts of interest to disclose.

